# Whole-transcriptome analysis reveals virulence-specific pathogen−host interactions at the placenta in bovine neosporosis

**DOI:** 10.3389/fimmu.2023.1198609

**Published:** 2023-07-14

**Authors:** Pilar Horcajo, Montserrat Coronado, Iván Pastor-Fernández, Esther Collantes-Fernández, Laura Rico-San Román, Armando Reyes-Palomares, Luis-Miguel Ortega-Mora

**Affiliations:** ^1^ SALUVET, Animal Health Department, Faculty of Veterinary Sciences, Complutense University of Madrid, Madrid, Spain; ^2^ Department of Biochemistry and Molecular Biology, Faculty of Veterinary Sciences, Complutense University of Madrid, Madrid, Spain

**Keywords:** *Neospora caninum*, transcriptome, bovine placenta, virulence, immune response

## Abstract

Research on bovine neosporosis has achieved relevant milestones, but the mechanisms underlying the occurrence of foetal death or protection against foetal death remain unclear. In a recent study, placentas from heifers challenged with the high-virulence isolate Nc-Spain7 exhibited focal necrosis and inflammatory infiltrates as soon as 10 days post-infection (dpi), although parasite detection was minimal. These lesions were more frequent at 20 dpi, coinciding with higher rates of parasite detection and the occurrence of foetal death in some animals. In contrast, such lesions were not observed in placentas from animals infected with the low-virulence isolate Nc-Spain1H, where the parasite was detected only in placenta from one animal at 20 dpi. This work aimed to study which mechanisms are triggered in the placentas (caruncles and cotyledons) of these pregnant heifers at early stages of infection (10 and 20 dpi) through whole-transcriptome analysis. In caruncles, infection with the high-virulence isolate provoked a strong proinflammatory response at 10 dpi. This effect was not observed in heifers infected with the low-virulence isolate, where IL-6/JAK/STAT3 signalling and TNF-alpha signalling *via* NF-κB pathways were down-regulated. Interestingly, the expression of E2F target genes, related to restraining the inflammatory response, was higher in these animals. At 20 dpi, more pronounced proinflammatory gene signatures were detectable in heifers infected with the high-virulence isolate, being more intense in heifers carrying dead fetuses. However, the low-virulence isolate continued without activating the proinflammatory response. In cotyledons, the response to infection with the high-virulence isolate was similar to that observed in caruncles; however, the low-virulence isolate induced mild proinflammatory signals at 20 dpi. Finally, a deconvolutional analysis of gene signatures from both placentome tissues revealed a markedly higher fraction of activated natural killers, M1 macrophages and CD8+ T cells for the high-virulence isolate. Therefore, our transcriptomic analysis supports the hypothesis that an intense immune response probably triggered by parasite multiplication could be a key contributor to abortion. Further studies are required to determine the parasite effectors that govern the distinct interactions of high- and low-virulence isolates with the host, which could help elucidate the molecular processes underlying the pathogenesis of neosporosis in cattle.

## 1 Introduction

*Neospora caninum* is an apicomplexan parasite responsible for a worldwide infectious disease that causes reproductive failure in cattle, called bovine neosporosis. Despite the substantial economic losses due to bovine neosporosis, very little is still known about the molecular mechanisms triggering abortion and foetal death in cows infected with *N. caninum* (1, 2). Two main mechanisms have been proposed to determine the outcome of gestation during infection: (i) foetal and/or placental lesions resulting from parasite multiplication and (ii) the shift from the prevailing Th2-anti-inflammatory response during gestation towards a Th1-proinflammatory response (3). However, other factors related to the parasite (intraspecific biological variability, parasite stage) or with the host (dam and foetal immunological status) have a marked influence on the dynamics of the parasite infection. This multifactorial scenario hinders the description of mechanisms that could trigger abortion (e.g., early impairment of local immune responses) or compromise foetal protection (e.g., expression of factors that modulate anti-inflammatory processes and tissue remodelling) (4, 5).

In previous studies, we have attempted to illustrate the complex host-parasite interactions that occur during infection through quantitative PCR, RNA-seq and proteomic approaches using specific bovine placental cell lines and macrophages cultured *in vitro* (6–9). Although the information provided by these works suggests different ways of interaction depending on the isolate, it neglects the intricate relationships between the different placental cell populations and the immune pressure imposed by resident immune cells. In ruminants, the main placental functions occur in the placentomes. These are formed by the foetal (cotyledon) and maternal (caruncle) compartments, whose circulatory systems are physically separated, adding some complexity to the response developed in the placenta. Such complexity is also increased by activity of innate immune cells such as macrophages, which might determine resistance against infection with *N. caninum* due to their ability to detect, phagocytose and eliminate parasites (10).

These limitations have been partially circumvented in recent years through *in vivo* trials performed in pregnant heifers (1). These experimental infections have shown that the outcome of infection is highly dependent on the moment of gestation when the infection occurs. In brief, pregnant cattle challenged at the first term of gestation (65–70 days) exhibit a rapid foetal death with a widespread parasite dissemination in placenta (11–15), while those infected at mid-gestation (110–140 days of gestation) can display either foetopathy or the birth of congenitally infected calves (15–17). Lastly, when infection occurs in the last third of gestation, all calves born healthy, but congenitally infected (12, 15).

Considering that naturally infected animal can give birth of healthy but congenitally infected animals or abort, and that abortion usually occur at the second term of gestation (18, 19), the mid-gestation models are regarded as a suitable tool for the study of the pathogenesis of bovine neosporosis. Recently, and using pregnant heifers experimentally challenged at day 110 of gestation, we found clear differences in the early dynamics of *N. caninum* infection –10 and 20 days post-infection (dpi)– between two different isolates, the highly virulent Nc-Spain7 and the low-virulence Nc-Spain1H (20). Just 10 days after infection, placentas from heifers challenged with the high-virulence isolate exhibited focal necrosis and inflammatory infiltrates, although parasites were detected only the 25% of the animals. These lesions were more frequent at 20 dpi, coinciding with higher rates of parasite detection and the occurrence of foetal death in the 40% of the animals. In contrast, placentas from animals infected with the low-virulence isolate Nc-Spain1H did not display any lesions or foetal death at 10 or 20 dpi, and the parasite was detected only in the 20% of the animals at 20 dpi (20). Previous results assessing the local immune response in these animals showed that the low-virulence isolate triggered the expression of pro-inflammatory cytokines, anti-inflammatory cytokines and other factors involved in the maintenance of ECM integrity at 10 dpi. However, as the infection progressed (20 dpi), a stronger Th1-based proinflammatory Th1-based response were observed in animals infected by the high-virulence isolate, that was stronger for some cytokines as IL-8 and TNF-α in placentomes from animals carrying non-viable fetuses (5).The placenta plays a key role in the pathogenesis of neosporosis: on the one hand, it acts as a physical and immunological barrier against infection, but on the other hand, it is able to trigger abortion through immune-mediated mechanisms (1). Indeed, previous studies that analyzed the expression of some cytokines at the materno-foetal interface after *N. caninum* infection, showed the up-regulation of proinflammatory cytokines (e.g. IFN, IL-12, TNF), often associated to foetal death, and also of Th2 and Treg cytokines (IL-4, IL-10), which are frequently associated to foetal survival (13–15, 21, 22). This immune stimulation is more accentuated at early gestation in the maternal side of the placenta, but in infections later on the gestation, the response increases in the foetal side of the placenta (13, 21, 22). Most of the previous work are limited to the study of a small number of cytokines, therefore to decipher the mechanisms triggered during the early host-parasite interactions that could be involved in the final outcome of the infection, we analyzed the whole transcriptome of caruncles and cotyledons from animals experimentally infected with high- (Nc-Spain7) and low-virulence (Nc-Spain1H) *N. caninum* isolates using a high-throughput technique for RNA sequencing (RNA-seq).

## 2 Materials and methods

### 2.1 Ethics statement

All protocols involving animals were approved by the Animal Research Ethics Committee of the Principado de Asturias, Spain (reference number PROAE 25/2016), following the proceedings described in Spanish and EU legislations (Law 32/2007, R.D. 53/2013, and Council Directive 2010/63/EU). All animals were handled in strict accordance with good clinical practices and all efforts were made to minimize suffering.

### 2.2 Animals, experimental design and sample collection

**Figure 1:**
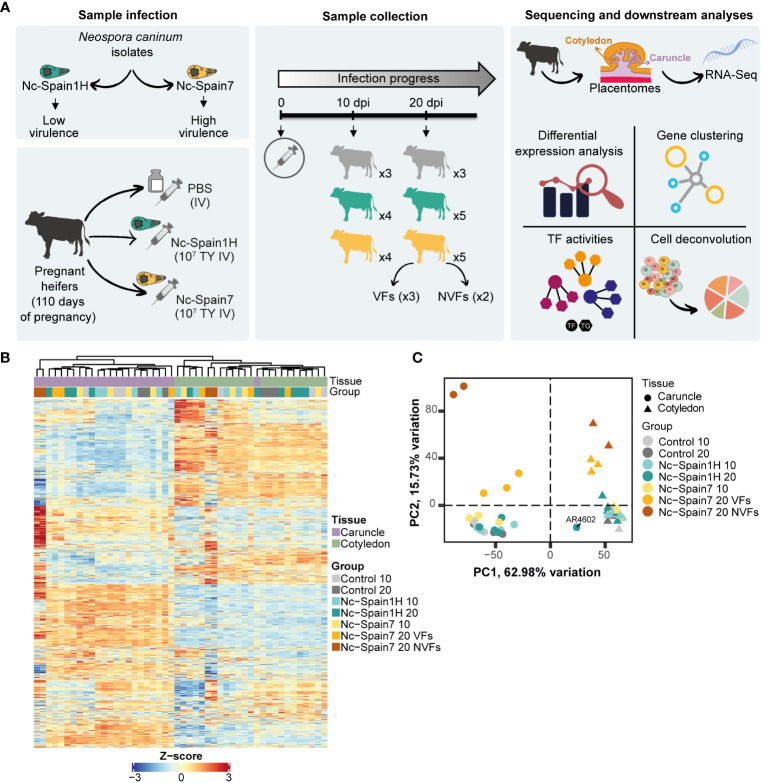
Experimental design for transcriptome analysis. **(A)** Overview of the experimental design. Colours represent the different groups of heifers: grey for control heifers inoculated with PBS, green for heifers infected with the Nc-Spain1H isolate and yellow for heifers infected with the Nc-Spain7 isolate. VFs, viable fetuses; NVFs, nonviable fetuses; TF, Transcription Factor; TG, Target Gene; TY, tachyzoites; IV, intravenously. **(B)** The heatmap represents the transcriptomic profile of each sample using Z scores from expressed genes in the caruncle (purple) and cotyledon (green). **(C)** Principal component analysis (PCA) of the top 5000 most variable genes from caruncle (circle) and cotyledon (triangle) samples across 10 and 20 dpi. Each point represents an RNA-Seq sample. *Neospora caninum*-infected samples and control samples are indicated by different colours. Outliers are labelled.

The experimental design has been described in detail previously (20). Briefly, 24 pregnant Asturian heifers were divided into 3 groups, two groups of 9 cows that were inoculated intravenously with 10^7^ tachyzoites of the Nc-Spain1H or Nc-Spain7 isolates at 110 days of gestation and a control group of 6 cows that were inoculated with phosphate-buffered saline at the same time. Half of the animals (3 from the control group and 4 from each infected group) were sacrificed at 10 dpi, and the remaining animals (3 from the control group and 5 from each infected group) were sacrificed at 20 dpi (**Figure 1A**). At this last time post-infection, foetal death was observed in 2 heifers infected with the Nc-Spain7 isolate.

For RNA-seq analyses, three random medial placentomes were carefully detached by hand, and maternal caruncles (CA) and foetal cotyledons (CO) were separated and placed in RNAlater (Thermo Fisher Scientific, Paisley, UK) and stored at −80°C until used for the analyses.

### 2.3 RNA extraction

Three medial CAs and COs from each animal were mixed separately for each tissue, and total RNA was extracted using the Maxwell^®^ 16 LEV simplyRNA Purification Kit, developed for the automated Maxwell^®^ 16 System (Promega, Madison, WI, USA), following the manufacturer’s recommendations.

### 2.4 Illumina library preparation and sequencing

The quantity and quality of the total RNA sample was determined by a Qubit^®^ RNA BR Assay kit (Thermo Fisher Scientific) and an RNA 6000 Nano Bioanalyzer 2100 Assay (Agilent, Santa Clara, CA, USA). The RNA-Seq library was prepared with the KAPA Stranded mRNA-Seq Illumina^®^ Platforms Kit (Roche Diagnostics Corporation, Indianapolis, IN, USA) following the manufacturer’s recommendations. Briefly, 500 ng of total RNA was used as the input material, the poly-A fraction was enriched with oligo-dT magnetic beads, and the mRNA was fragmented. Strand specificity was achieved during second strand synthesis performed in the presence of dUTP. The blunt-ended double-stranded cDNA was 3´ adenylated, and Illumina platform-compatible adaptors with unique dual indices and unique molecular identifiers (Integrated DNA Technologies, Inc., Coralville, Iowa, USA) were ligated. The ligation product was enriched with 15 PCR cycles, and the final library was validated on an Agilent 2100 Bioanalyzer with the DNA 7500 assay.

The libraries were sequenced on a NovaSeq 6000 (Illumina, San Diego, CA, USA) with a read length of 2x101 bp following the manufacturer’s protocol for dual indexing. Image analysis, base calling and quality scoring of the run were processed using the manufacturer’s software Real Time Analysis (RTA v3.4.4), followed by generation of FASTQ sequence files.

RNA-Seq raw reads were preprocessed using the Fastp tool (23) to filter out low-quality reads and adapter sequences. Quality control of fastq files was performed using FastQC software (v0.11.4) (24) before and after trimming. Then, the filter paired-end reads were mapped against the *Bos taurus* reference genome (ARS-UCD1.2) provided by the ENSEMBL database release 103 using STAR (v2.7.0) (25), and gene-level abundances were estimated using the genecounts option from STAR software.

### 2.5 Differential gene expression analysis

Differential expression analysis of RNA-Seq was performed separately for caruncle and cotyledon samples using the DESeq2 package (v1.30.1) (26). Genes with low expression (those genes with an average expression across all samples from the same tissue less than 1 read) were removed before the gene expression analysis, leaving 19392 and 18468 unique genes for the caruncle and cotyledon samples, respectively. Expression data were normalized using the variance stabilization transform (VST) for exploratory data analysis using principal component analysis (PCA) and hierarchical clustering. PCA of the top 5000 most variable genes was performed by using the PCAtools package (v2.2.0) (27) from Bioconductor. Differential expression analysis was performed by comparing Nc isolate-infected samples and all control samples (Nc-Spain1H compared to the control and Nc-Spain7 compared to the control) at 10 and 20 dpi. For each analysis, genes with a 5% false discovery rate (FDR) and at least 30 raw read counts in 70% of the samples from a group included in the contrast were considered differentially expressed genes (DEGs). To calculate the similarity between the DEGs obtained from the different contrasts, we used the overlap_coefficient function of the Genetonic package (v1.2.0) (28). Briefly, the overlap coefficient algorithm calculates the similarity between two sets of features by using the intersection of these two sets and dividing it by the smaller set.

### 2.6 Functional and gene set enrichment analysis

We used the clusterProfiler package (v3.18.1) (29) to perform Gene Ontology (GO) biological process (BP) and Kyoto Encyclopedia of Genes and Genomes (KEGG) enrichment analyses using DEGs from each comparison and expressed genes as the background list. We used the biomaRt package (v2.46.3) (30) to build a GO annotation for biological processes associated with ENSEMBL gene IDs. org.Bt.eg.db (v3.12.0) (31) from Bioconductor were used to retrieve KEGG annotations from the Bos taurus genome. Enrichment terms with at least 3 genes and P values adjusted by FDR < 0.05 were regarded as significant enrichment terms.

To reduce the term lists from GO enrichment analysis, we calculated the semantic similarity between pairwise GO terms and clustered them using the rrvgo package (v1.2.0) (32) from Bioconductor. Cluster trees were cut with a threshold between 0.6 and 0.9 depending on the number of terms, and the more significant GO enriched term (that with a low FDR value) was selected as a representative term.

Gene Set Enrichment Analysis (GSEA) (33) was also performed using the clusterProfiler package. We conducted GSEA using a gene list ranked by the stat parameter from DESeq2 and the hallmark gene set from the Molecular Signatures Database (MSigDB) (34) (msigdbr R package v7.4.1) (35). We selected pathways with an FDR value < 0.1 as significant.

### 2.7 Clustering analyses

Expressed genes were used to analyze the dynamics of each isolated infection. VST-normalized expression was scaled to the z score, and the mean of the genes of each group was used for clustering. Genes were clustered using the Partition Around Medoids (PAM) method. To validate the cluster results, we calculated the silhouette coefficient and visualized it using the fviz_silhouette function from the factoextra package (v1.0.7) (36). We also performed functional enrichment analysis for each subset of genes using the GO BP annotation as described above.

### 2.8 Deconvolution analysis

To estimate the percentage of immune cell populations in caruncle and cotyledon RNA samples, we used the CIBERSORTx algorithm (37). Gene expression was normalized to transcript per million (TPM), and ENSEMBL ID from the *Bos taurus* genome was converted to human orthologues using biomaRt (v2.46.3). To select the best orthologue pairs in case there was more than one human orthologue for different cow genes, we ranked the genes by their human gene order conservation score, whole genome alignment coverage and average between target and query percent identicality and selected orthologues with a homology orthology confidence equal to 1. After that, ENSEMBL ID was converted to gene symbols to create the mixture file. As the signature matrix, we used the leukocyte signature matrix (LM22) available in CIBERSORTx, which has signature genes from 22 different immune cell types. To run CIBERSORTx, we used B-mode batch correction with the gene expression profile (GEP) file from LM22, included in their website, 100 permutations and absolute mode.

### 2.9 Transcription factor activity analysis

We used the decoupleR package (v2.3.2) (38) from Bioconductor to infer the transcription factor (TF) activities in two different ways, using the gene expression matrix and using the log2 fold change (FC) obtained from differential expression analysis. Gene expression was normalized using the VST function from the Deseq2 package. To infer TF activities, we used DoRothEA (39) regulons from the human genome with the confidence level ABC. Gene names from the *Bos taurus* genome in the normalized gene expression matrix were converted to human orthologues as explained before. We used the Weighted Mean method to infer activities using the run_wmean function with 1000 permutations and a minimum number of targets per TF = 5, and we selected norm_wmean activities.

### 2.10 Statistical analysis and graphics

All statistical analyses were conducted in R (v4.0.3) (40). The ComplexHeatmap (v2.6.2) (41) and ComplexUpset (v1.3.3) (42, 43) packages were used to represent the heatmaps and upset plots, respectively. The overlap coefficient plot was generated using the corrplot package (v0.92) (44). The other plots were generated using the ggplot2 package (v3.3.6) (45).

## 3 Results

### 3.1 Placental tissue and infection status dominate transcriptome remodelling

To study the placental responses to infection by different *N. caninum* isolates at the transcriptional level, we performed exploratory data analysis of RNA-Seq data from caruncles and cotyledons obtained from heifers infected with the high-virulence isolate Nc-Spain7 (*n* = 9) and the low-virulence isolate Nc-Spain1H (*n* = 9) and noninfected heifers (*n* = 6) (**Figure 1A**). A total of 48 samples were profiled with an average of 34.1 million reads per sample (**Supplementary Figure 1A**). From all samples, between 75 and 96% of reads were mapped against the reference genome (**Supplementary Table 1**). Caruncles and cotyledons exhibit clearly distinguishable transcriptomes (**Figure 1B**) and samples from heifers where foetal viability was compromised show recognizable transcriptomic signatures (**Figure 1B**). Pearson correlation coefficients revealed two main clusters of samples corresponding each to the tissue of origin (i.e. cotyledon or caruncle) (**Supplementary Figure 1B**). This prominent separation between cotyledon and caruncle samples indicates the existence of a specific transcriptional program for each component of the placenta.

Principal component analysis of most variable genes revealed clusters of samples according to the placental tissue, the isolate used for experimental infection and the culling date. As expected, principal component 1 (PC1) showed that caruncle- and cotyledon-specific gene signatures accounted for the main source of variation (62.98%). In addition, principal component 2 (PC2) revealed clusters of samples corresponding to animals infected by the high-virulence isolate at 20 dpi (15.73% variation) (**Figure 1C**). We also found a separation in PC2 between samples of Nc-Spain7-infected animals from heifers that had suffered foetal death (NVFs, nonviable fetuses) and those whose fetuses were still alive (VFs, viable fetuses) at 20 dpi (**Figure 1C**). However, we did not find an obvious separation between caruncles or cotyledon samples from uninfected animals versus those infected with the low-virulence isolate (Nc-Spain1H) at any dpi or with the high-virulence isolate (Nc-Spain7) at 10 dpi. During these analyses, we detected an outlier (AR4602) among the caruncle samples infected with the Nc-Spain1H isolate that clustered with other cotyledon samples both in the PCA (**Figure 1C**) and in the heatmap of expressed genes (**Figure 1B**). We attributed this to an incorrect separation of the caruncle and cotyledon during mechanical placentome detachment, and consequently, this sample was removed for further analysis.

Transcriptomes resulting from uninfected animals were markedly distinguishable by their cotyledonary or caruncular origin but not by the day of euthanasia (10 or 20 dpi) (**Figure 1B**). Hence, regardless of the time of euthanasia, all control samples collected from the same type of tissue were considered as a single condition for further analyses. In addition, the heatmap revealed a lower correlation of the NVF group compared to other groups, which was more noticeable in caruncle samples. Therefore, NVF samples from the caruncle and cotyledon were discarded for differential expression analysis to avoid the potential interference of the foetal death process on the observed transcriptomic modifications.

### 3.2 Transcriptome remodelling robustly increases in caruncles during the infection course and in an isolate-dependent manner

**Figure 2:**
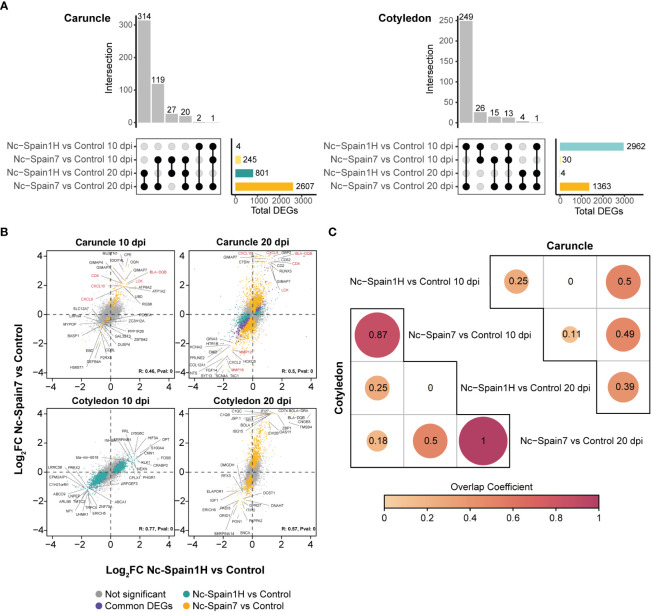
Transcriptomic analysis of *N. caninum* isolate-infected samples and noninfected samples. **(A)** The UpSet plot shows the intersection of differential expressed genes (DEGs) obtained from the differential expression analysis of each contrast from the caruncle (left) and cotyledon (right). Vertical bars show the number of common DEGs between different contrasts, according to the black circles connected. Horizontal bars show the total number of DEGs in each comparison. Genes with a 5% FDR were considered DEGs. **(B)** Pearson Correlation of log2-fold change between high- and low-virulence infections. Each dot represents a gene, and the top 15 genes with the top absolute log2-fold change significant in at least one contrast are labelled. Green represents DEGs between the Nc-Spain1H-infected and control samples, yellow represents DEGs between the Nc-Spain7-infected and control samples, purple represents common DEGs between the two contrasts and grey represents no significant genes in any comparisons. **(C)** Corrplot shows the overlap coefficient between DEGs from the Nc-Spain1H-infected samples compared to the control samples and the Nc-Spain7-infected samples compared to the control samples at 10 and 20 dpi separately in the caruncle (upper) and cotyledon (lower).

*Neospora caninum* induced robust transcriptomic changes in placentomes during infection, which were more prominent for the high-virulence isolate. In caruncles, subtle differences in gene expression levels were found at the early stages of the infection (10 dpi) and, more particularly, in the case of the low-virulence isolate (Nc-Spain1H) where only 4 genes were differentially expressed (e.g., *SLC12A7, KIFC3, LRFN4* and *TOLLIP*) with lower mean expression levels across infected animals than noninfected animals (**Figure 2A**). In the case of the high-virulence isolate (Nc-Spain7), a total of 245 (DEGs resulted from the comparison between infected and noninfected animals, where *TOLLIP* also had a lower mean expression across infected samples (**Figure 2A**). At the later stage of the infection (20 dpi), the transcriptomic differences were more pronounced for both isolates, accounting for 801 and 2,607 DEGs for the Nc-Spain1H and Nc-Spain7 isolates, respectively. **Figure 2A** shows the number of DEGs and resulting intersections between tested contrasts, and these are summarized in **Supplementary Table 2**.

Despite the quantitative differences in the differential expression analysis between isolates, we found a higher expression of some genes related to immunity (e.g., *LCK, CXCL9, CXCL10, CD4, BLA-DQB*) at 10 and 20 dpi and a decreased expression of genes coding for matrix metalloproteinases (e.g., *MMP12* or *MMP16*) at 20 dpi in all samples infected with the Nc-Spain7 or Nc-Spain1H isolates. Additionally, to assess the relationship between the infection status (uninfected versus infected) and the virulence of the isolate, we calculated pairwise correlations of gene expression changes from each contrast. Log_2_ FC from contrasts between the Nc-Spain7- and Nc-Spain1H-infected animals compared to the controls resulted in weak positive correlations at 10 dpi (R = 0.46, P value = 0) and 20 dpi (R=0.5, P value = 0) (**Figure 2B**). The resulting correlations suggest a dominance of gene signatures that are mainly associated with infection status, but there are some differences that could be explained by other experimental factors, such as the mechanisms of the virulence of each isolate (**Figure 2B**).

### 3.3 Transcriptomic changes induced by low- and high-virulence isolates were similar in cotyledons

In cotyledons, the number of DEGs between infected and noninfected animals at 10 dpi was notably higher in those infected with the low-virulence isolate (2,962 DEGs) than in those infected with the high-virulence isolate (30 DEGs). However, despite these differences, the fold change of genes between the infected and noninfected samples exhibited a strong positive correlation (R=0.77, P value = 0; **Figure 2B**), which indicates a robust resemblance of the isolate-induced changes in cotyledons. During the late stage of the infection (20 dpi), gene expression differences were detected only between the heifers infected with the high-virulence isolate and the noninfected animals (1,363 DEGs), instead only 4 DEGs were detected between low-virulence isolate and the noninfected animals (**Figure 2A**). Again, a positive correlation was also found for fold change values from both groups at this stage of infection (R=0.57, P value = 0) (**Figure 2B**).

Therefore, although differential expression analyses showed substantial differences between isolates in cotyledons, their gene expression patterns were strongly correlated with the infection status. This finding suggests that the isolate used for infection has less influence on the transcriptomic changes in cotyledons than in caruncles. Indeed, there was no correlation between the fold change values of the equivalent contrast in the caruncle and cotyledon (**Supplementary Figure 2**). This finding is in accordance with the different responses observed in the maternal and foetal placenta after *N. caninum* infection. To evaluate the degree of similarity between the list of DEGs derived from each contrast, we computed the overlap coefficient between contrasts that were very high in cotyledons between isolates according to the course of the infection (**Figure 2C**). Detailed results of all differential expression analyses in cotyledons can be found in **Supplementary Table 3**.

### 3.4 Proinflammatory signals induced by low- and high-virulence isolates are opposite in caruncles and differentially activated in cotyledons

To investigate the underlying mechanisms during responses against the high- and low-virulence isolates of *N. caninum*, we performed functional enrichment analyses for GO and KEGG annotations. Overrepresentation analysis of BP GO terms and KEGG pathways by querying the lists of DEGs revealed an enrichment in processes related to the immune system and defence response, either in caruncles or cotyledons, eliciting different immune processes according to the dpi or the isolate. For instance, the contrast between the Nc-Spain7-infected and noninfected animals in caruncle revealed the enrichment of several GO terms related to T-cell differentiation at 10 dpi, but at 20 dpi, an enrichment of immune processes involved in the regulation of cytokine production and stimulation was observed (**Supplementary Figure 3A**). Although there was no enrichment of GO terms among the DEGs between the Nc-Spain1H-infected samples and controls in caruncles (**Supplementary Figure 3A**), the analysis using annotation from KEGG pathways revealed an enrichment of processes related to T-cell differentiation at 20 dpi, such as “Th1 and Th2 cell differentiation” and “Th17 cell differentiation” (**Supplementary Figure 3B**). Interestingly, in cotyledons, a robust enrichment of proinflammatory processes was found only with the high-virulence isolate at 20 dpi (**Supplementary Figure 3**). No significant enrichment was found in cotyledons of low virulence isolate-infected animals, which is striking due to the high number of DEGs at 10 dpi.

**Figure 3:**
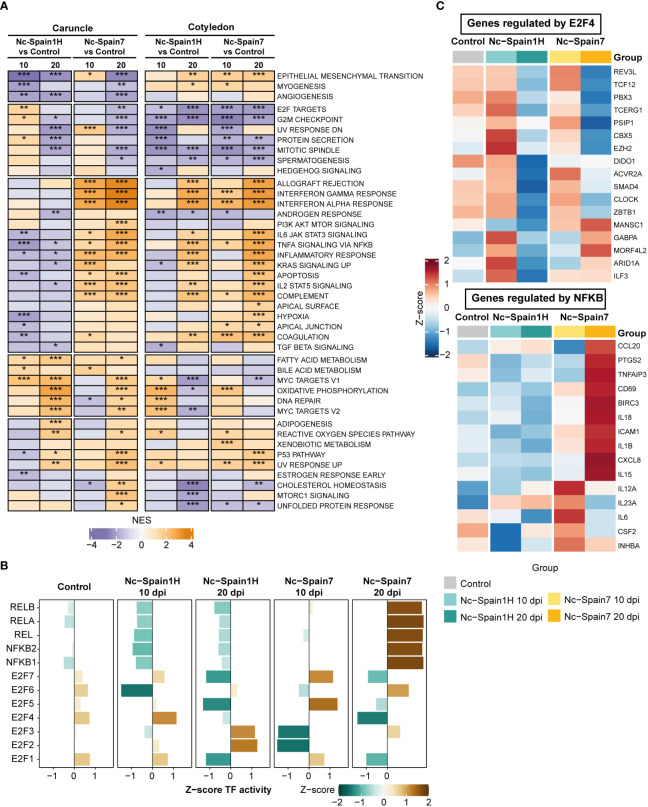
Functional analysis of high- and low-virulence infected samples compared to control samples. **(A)** A heatmap shows the normalized enrichment scores (NES) of GSEA for hallmark gene sets using differential gene expression from the Nc-Spain1H-infected samples compared to the control samples and the Nc-Spain7-infected samples compared to the control samples separately by culling date and tissue. Pathways enhanced (NES >0) in the *N. caninum*-infected samples are in orange, and pathways decreased (NES < 0) in the *N. caninum*-infected samples are in purple. Adjusted *p* values are represented by stars, *** p.adjust < 0.001, ** p.adjust < 0.01, * p.adjust < 0.1. **(B)** Bar plots represent the Transcription factor (TF) activity of the E2F and NF-κB TF families calculated using gene expression from the *N. caninum* isolate-infected and control samples in caruncles. Axis x represents the Z score of mean TF activity by group. **(C)** Heatmaps show the expression levels of selected gene targets of E2F4 (upper) and NFKB (lower) in caruncle tissue separately in the control, Nc-Spain1H-infected and Nc-Spain7-infected samples.

To further explore the functional implications of the transcriptional differences induced by *N. caninum* infection in placentomes, we performed a GSEA using a curated hallmark gene set database. GSEA revealed deeper details of transcriptomic changes found in each placental tissue according to dpi or isolate. Overall, functional enrichments were markedly different between isolates in the caruncle compared to the cotyledon. One of the most significant differences was the downregulation of hallmark genes associated with the inflammatory response, IL-6/JAK/STAT3 signalling, tumor necrosis factor (TNF)-alpha signalling *via* NF-κB and coagulation in caruncles infected by the low-virulence (Nc-Spain1H) isolate (**Figure 3A**). Conversely, these gene sets and others, such as IFN-γ and IFN-α responses, IL2-STAT5 signalling, and complement, were upregulated early in caruncle samples infected by the high-virulence isolate (**Figure 3A**). In addition, these proinflammatory gene sets were consistently upregulated in caruncles during the later stage of infection by the high-virulence isolate. However, the proinflammatory immune response was not induced in any of the stages of the infection with the low-virulence isolate.

In cotyledons, genes associated with the IFN response, TNF-alpha signalling *via* NF-κB, complement and coagulation were only stimulated by the high-virulence isolate at the earliest stage of infection. However, at 20 dpi, both isolates induced a strong proinflammatory response in this tissue (**Figure 3A**). Nevertheless, the comparison between isolates showed that this response was stronger in the animals infected with the high-virulence isolate (**Supplementary Figure 4A**).

Interestingly, a slight but significant upregulation of target genes of E2F transcription factors and pathways related to the cell cycle (e.g., G2M checkpoint) was observed only in caruncles infected with the low-virulence isolate (**Figure 3A**). Notably, although E2F target genes and the G2M checkpoint were downregulated in cotyledons from all infected samples, we observed strong differences between samples infected by the high- and low-virulence isolates (**Supplementary Figure 4A**, **Supplementary Tables 4, 5**).

Finally, some essential physiological processes (e.g., cholesterol homeostasis, epithelial mesenchymal transition, apoptosis and hypoxia) revealed discordant activities between isolates in caruncle samples along the infection course. In contrast, genes related to oxidative phosphorylation had a very similar profile in infections by both isolates. All the results derived from the functional enrichment analysis are detailed in **Supplementary Tables 6**, **7**.

### 3.5 Increased E2F4 activity in samples infected by the low-virulence isolate in caruncles

Given the differences in host responses to infection of the two isolates in the caruncle, we performed an analysis to infer TF activity based on the expression levels of their target genes in caruncle samples. Focusing on the enriched TF families, such as NF-κB and E2F, we identified higher activity of the NF-κB family (e.g., NFKB1, NFKB2, REL, RELA, RELB) in samples from the heifers infected with the high-virulence isolate at 20 dpi and lower activity for the Nc-Spain1H-infected samples at 10 dpi (**Figure 3B**). These samples also displayed increased E2F4 activity, which was decreased in samples from the animals infected by the high-virulence isolate (**Figure 3B**). To determine whether TF activity varied using the results of differential expression analysis, we also calculated TF activity using log2FC values from comparisons between isolates at 10 dpi in caruncles and selected the top 15 TFs with the highest activity in each isolate. These results also revealed an increase in the activity of TFs with an important role in the immune response against pathogens such as STAT1, STAT4, and NFKB1 in the samples infected with the Nc-Spain7 isolate. In addition, the Nc-Spain1H-infected samples showed an increase in E2F4 activity, among others (**Supplementary Figure 4B**).

Considering that previous studies have demonstrated an inhibition of the NF-κB pathway associated with the expression of E2F3/E2F4-regulated genes upon *Toxoplasma gondii* infection (46), we examined the expression of selected E2F4 target genes and observed that some genes, such as *EZH2, CBX5, GABPA, ARID1A* and *ILF3*, showed higher expression in the Nc-Spain1H-infected samples at 10 dpi (**Figure 3C**). In particular, *EZH2* has been shown to inhibit the expression of NF-κB target genes. Accordingly, we found that the expression of NF-κB target genes inhibited by *EZH2* is decreased in the Nc-Spain1H-infected samples. However, their expression was elevated in the Nc-Spain7-infected samples, especially at 20 dpi (**Figure 3C**). These findings are consistent with the results of the TF activity analysis (**Figure 3B**).

### 3.6 Quantitative real-time PCR (qPCR) recapitulates transcriptomic differences detected by RNA-Seq

RNA-Seq results were validated using qPCR data of genes related to immune and inflammatory responses that were analysed previously (5). More specifically, the FC value from qPCR data on each gene was calculated as the difference between the Ct value from each sample and the Ct value from the control samples. The resulting value was used as ΔΔCt, and 2^–ΔΔCt^ was used to compare with the fold change value obtained from the RNA-Seq analysis. RNA-Seq and qPCR values were significantly positively correlated in most cases in caruncle as well as cotyledon samples. Only the contrast between the Nc-Spain1H-infected samples and the control samples at 10 dpi in cotyledons showed little correlation and a nonsignificant P value (**Supplementary Figures 5A, B**).

### 3.7 Cluster analysis reveals gene signatures specifically related to foetal death during infection with the high-virulence isolate

To investigate the relationship of the infection with foetal death, we analysed gene signature patterns from noninfected and Nc-Spain7-infected animals by classifying in independent groups those heifers euthanized either at 10 or 20 dpi and carrying viable or nonviable fetuses.

**Figure 4:**
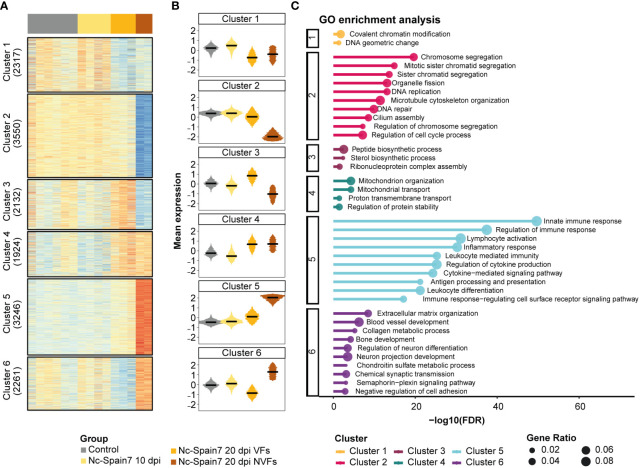
Gene expression dynamics from noninfection to foetal death in caruncle tissue of animals infected by the high-virulence isolate Nc-Spain7. **(A)** Heatmap of normalized gene expression (Z score) for the control and Nc-Spain7-infected samples. Number of genes on each cluster indicated on the left annotation. **(B)** Boxplots represent the mean expression of genes under each condition. **(C)** Top 10 most significant terms (lower adjusted P value) of each cluster. Axis x represents the -log10 adjusted P value, and circle size represents the gene ratio of each enriched term.

In the caruncle, a total of 15,430 genes expressed across samples were partitioned into 6 clusters corresponding to specific expression patterns (**Figures 4A, B**). Overrepresentation analysis of GO terms for each cluster was carried out to identify biological processes that might be involved in the progression and severity of infection to foetal death (**Figure 4C**, **Supplementary Table 8**). Cluster 2 revealed gene signatures that decreased their expression during the infection until foetal death had occurred, revealing several genes associated with the cell cycle. Conversely, Cluster 5 showed an increased expression of genes during the infection course and a significant enrichment of terms related to immune system processes. Several genes related to mitochondrial organization and transport were upregulated in Nc-Spain7 infection at 20 dpi, as shown in Cluster 4 (**Figure 4C**). In addition, 2 divergent patterns between the VF and NVF samples (Cluster 3 and Cluster 6) were identified. Samples from the heifers carrying VFs displayed an increase in genes related to peptide and sterol biosynthetic processes, and these were inhibited in samples from the heifers carrying NVFs (Cluster 3). In addition, the VF samples showed a decrease in genes related to extracellular matrix organization and tissue development, and these genes were upregulated in the NVF samples (Cluster 6).

**Figure 5:**
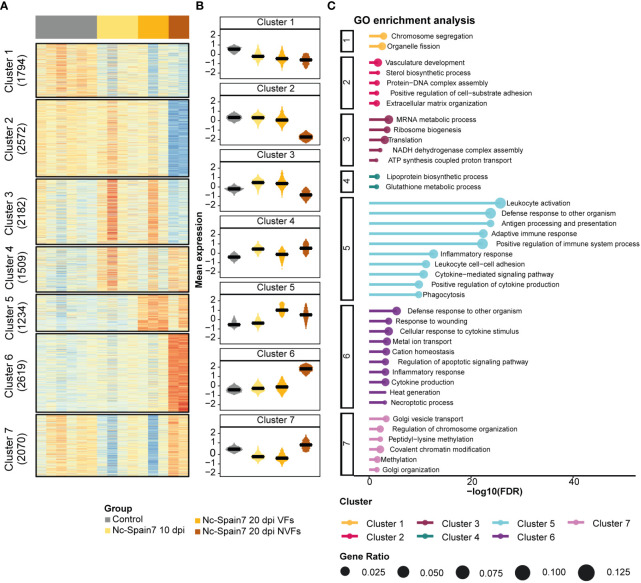
Gene expression dynamics from noninfection to foetal death in cotyledon tissue of animals infected by the high-virulence isolate Nc-Spain7. **(A)** A heatmap of normalized gene expression (Z score) for the control and Nc-Spain7-infected samples. Number of genes on each cluster indicated on the left annotation. **(B)** Boxplots represent the mean expression of genes under each condition. **(C)** Top 10 most significant terms (lower adjusted P value) of each cluster. Axis x represents the -log10 adjusted P value, and circle size represents the gene ratio of each enriched term.

In the cotyledons, 13,980 gene signatures were clustered into 7 subsets of genes (**Figures 5A, B**; **Supplementary Table 9**). Clusters 1 and 2 were downregulated as the infection progressed until foetal death was observed. However, interestingly, Cluster 2 revealed a specific downregulation of genes involved in extracellular matrix organization and vasculature development in the NVF samples. In contrast, progression of *N. caninum* infection induced an upregulation of genes associated with ribosome biogenesis and translation (Cluster 3) and immune response (Clusters 5 and 6) (**Figure 5C**). However, decreased expression of genes associated with ribosome biogenesis and translation (Cluster 3) and with the activation of the inflammatory immune response (Cluster 5) was observed once foetal death was evident, and this was also associated with increased expression of apoptotic and necroptotic processes (Cluster 6) (**Figure 5C**).

### 3.8 Deconvolution analysis reveals different fractions of immune cell contributions during *N. caninum* infection in caruncle and cotyledon tissues

To elucidate the immune cell types involved in *N. caninum* infection for each isolate, we employed the CIBERSORTx tool to perform a deconvolution analysis of bulk RNA samples (37).

**Figure 6:**
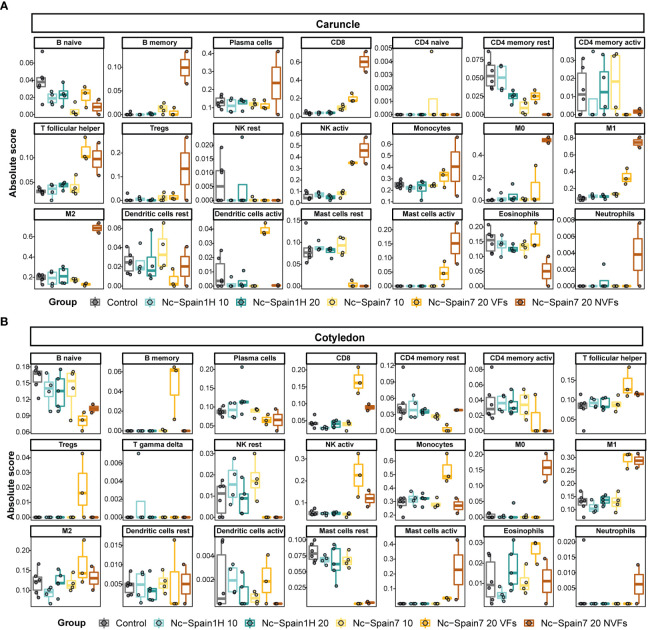
Deconvolution results of the placentome using the CIBERSORTx tool. Boxplots represent the absolute score of each immune cell type in the LM22 signature matrix obtained in the deconvolution analysis in each sample group separated for caruncle **(A)** and cotyledon **(B)** tissues.

Deconvolution of caruncle Nc-Spain7 NVF samples revealed an increase in immune cell types such as monocytes, macrophages (M0, M1 and M2), activated NK cells, CD8 cells, B memory cells, T follicular helper cells and activated mastocytes. Most of these cell populations were also increased in the Nc-Spain7 VF samples, except for the M2 cell fraction, which was lower in the Nc-Spain7 VF samples than in the samples from animals infected with the Nc-Spain1H isolate. We also found that eosinophils and B naïve cells had a lower representation on the Nc-Spain7 NVF samples (**Figure 6A**).

The deconvolution analyses carried out in samples from cotyledons demonstrated that the Nc-Spain7 VF samples had an increase in the proportion of macrophages (M1 and M2), activated NK cells, B memory cells, T follicular helper cells, Tregs and CD8 cells, while the Nc-Spain7 NVF samples had a higher proportion of M0 macrophages and activated mastocytes (**Figure 6B**).

## 4 Discussion

Here, for the first time, we use a pregnant bovine model of experimental neosporosis at mid-gestation to study whole-transcriptome modifications in the placenta during early infection with virulent and nonvirulent isolates of *N. caninum*. Pregnant heifers were sacrificed at 10 and 20 dpi upon inoculation with high-(Nc-Spain7) or low-(Nc-Spain1H) virulence isolates to analyse the host-parasite interactions that occur prior to abortion, which often occurs 30 days after infection. Since the host-parasite interactome in the placenta may play a key role during pathogenesis (47–49), we devised a comparative transcriptome analysis focused on the foetal-maternal interface from heifers infected by both *N. caninum* isolates.

Since the foetal-maternal interface is composed of the interdigitation of foetal (cotyledon) and maternal (caruncle) parts, both tissues were considered separately in our experimental design. As expected, our exploratory analyses revealed two well-defined and different transcriptional programs corresponding to caruncle and cotyledon samples. This difference between tissues has been recently corroborated by Diniz et al. (50), who found 2,654 genes and pathways, such as nutrient transport, tissue differentiation and remodelling, and immune tolerance, that were differentially regulated between caruncles and cotyledons from healthy nulliparous beef heifers. These tissue-specific transcriptional differences persist even in the presence of different isolates, indicating that the primary driver of variation lies within the tissues themselves. However, it is important to note that our results also revealed altered isolate-specific processes in both tissues, suggesting an interplay between tissue-specific programs and isolate-induced responses.

Differential expression analysis between samples from the infected and noninfected heifers revealed positive correlations of their gene signatures in the caruncle and cotyledon according to the dpi, which reflects a similar interaction of both isolates with the host. This correlation was especially striking at the foetal part of the placenta and had been previously observed *in vitro* using bovine trophoblast cells (7). These results could reflect the greater necessity of *N. caninum* to tightly regulate the maternal immune system to be able to cross the placenta and reach the fetus, where the lack of mature immune pressure would allow the parasite to replicate easily. The higher transcriptomic differences observed between isolates in the caruncles could account for the different infection outcomes triggered by each isolate. A common signature of *N. caninum* infection was the enhancement of oxidative phosphorylation and mitochondrion-related processes at 10 dpi in cotyledon samples and at 20 dpi in caruncle samples. Indeed, the interaction of *T. gondii* and *N. caninum* with the mitochondria has been extensively demonstrated (51, 52) and corroborated in this study in the case of *N. caninum*. These results indicate that the activation of the mitochondrial respiratory chain is part of the early host responses against infection, as this is a major source of reactive oxygen species (ROS). This hypothesis is robustly supported by the enhanced reactive oxygen species pathways that we observed both in caruncles and cotyledons at the same times after infection. In a comparison of both isolates, the oxidative phosphorylation process appeared to be more highly enhanced by the low-virulence isolate at 10 dpi. This phenomenon may allow the host to control the Nc-Spain1H infection earlier, as suggested previously (5). This observation agrees with the higher impairment of ROS production found in bovine macrophages infected by the high-virulence isolate compared to those infected with the low-virulence isolate (53). These results could be related to the higher abundance of the putative glucose-6-phosphate dehydrogenase (G6PD, NCLIV_000940) protein of *N. caninum* in high-virulence isolates (9, 54). This enzyme contributes to maintaining the redox balance (55), which could help parasites survive and proliferate in host cells. In addition, a recent study demonstrated a novel immune evasion mechanism in *N. caninum* based on the promotion of host mitophagy (56). This mechanism could impair the production of ROS and attenuate the production of proinflammatory cytokines. This finding opens a new line of research to determine if isolates that display differences in ROS production and activation of proinflammatory responses are also able to modulate the mitophagy process in an isolate-dependent manner.

Regarding differences found between isolates, the immune response deserves special attention. Our results highlighted that infection with the high-virulence isolate Nc-Spain7 induced a stronger immune response both in caruncles and cotyledons, although these differences were especially notable in the maternal side of the placenta. These results agree with previous studies that have shown upregulation of proinflammatory response in placenta, mainly in maternal part, after *N. caninum* infection with high-virulence isolates at different stages of infection (14, 21, 22, 57). The overexpression of genes related to TNF-alpha signalling *via* NF-κB, IL-6/JAK/STAT3 signalling, IL-2/STAT5 signalling, IFN-γ and IFN-α responses in caruncles demonstrates the activation of a strong proinflammatory response after infection with the high-virulence isolate, which was not observed in heifers infected with the low-virulence isolate. Indeed, GSEAs showed a downregulation of gene sets such as TNF-alpha signalling *via* NF-κB, IL-6/JAK/STAT3 signalling and inflammatory response after infection with the low-virulence isolate at an early stage of infection. These results contrast with the stronger proinflammatory response described for the low-virulence isolate at 10 dpi when a some cytokines were analysed by qPCR (5). This discrepancy between studies likely reflects the huge differences between qPCR approaches and RNA-seq in terms of power and resolution. The results obtained through the full transcriptome sequencing performed in this study are based on the expression of more than thirteen thousand genes, by contrast, in qPCR-based studies only a few genes are analysed. Therefore, the results of the present study show a more realistic picture of the interactions at the placentome level during early infection. In this study the transcriptome of caruncles obtained from animals infected with the low-virulence isolate at 10 dpi was very similar to that observed from the same tissues in noninfected animals, with only 4 genes found to be differentially expressed. Interestingly, *TOLLIP* was downregulated in both isolates. This gene has been associated with an anti-inflammatory effect by dampening TLR-mediated inflammation and therefore suppressing the expression of inflammatory mediators such as TNF-α, IL-6 and IL-1β, among others (58, 59). This phenomenon may be an explanation for the significant overexpression of IL-6 observed in the Nc-Spain7-infected samples. *Leishmania donovani* has been suggested to exploit TOLLIP for its own establishment (60), and specific studies are still necessary in *N. caninum* to determine its role in parasite biology. The effect of the downregulation of this gene stimulating the immune response was not observed in the Nc-Spain1H-infected animals. The lack of proinflammatory response by the Nc-Spain1H isolate in the caruncle at 10 dpi could be related to the overexpression of E2F3/E2F4-regulated genes observed in infected heifers. The E2F-regulated gene *EZH2* could be responsible for the negative regulation of NF-kB activity during *T. gondii* infections, which contributes to the host immune equilibrium and parasite persistence in mice (46). Here, we observed that *EZH2* was only upregulated in heifers infected by the low-virulence isolate, which could be considered an isolate-specific regulation that provides an evolutionary advantage to the parasite. This regulation would prevent an exacerbated immune response, avoiding placental damage and abortion and promoting parasite persistence through harmless transmission to successive generations. In *T. gondii*, *EZH2* gene activation seems to be induced by the TEEGR effector (46). However, this effector is poorly conserved in the *N. caninum* genome (46), indicating the need for future exploration of the genes responsible for this phenotype.

Notably, as early as 10 dpi, genes related to apoptosis are overexpressed in the caruncles of animals infected with the high-virulence isolate but downregulated in those infected by the low-virulence isolate. The modulation of apoptotic processes has been extensively described in *N. caninum* and *T. gondii* (6, 61–63), but its biological importance remains unclear. Histological examination of placentas from heifers infected with the high-virulence isolate showed areas of focal necrosis that were not found in samples from animals infected with the low-virulence isolate (17, 20). Although both processes are different, they could be related, as necroptosis is defined as a programmed form of necrotic death and can be ignited by the same death signals that induce apoptosis but also by pathogen recognition receptors, including Toll-like receptors and NOD-like receptors (64, 65). In any case, it is likely that excessive cellular death had a negative impact on the microenvironment of the maternal–foetal interface and could help to trigger foetal death (66).

Differences between high- and low-virulence isolates are much more marked as the infection progresses. At 20 dpi, the Nc-Spain7-infected animals displayed a stronger induction of the immune response and apoptosis, whereas the immune response was much more moderate and controlled in the animals infected with low-virulence isolates. This finding is in line with previous studies that showed that the Nc-Spain1H isolate is not able to grow exponentially *in vitro* in a bovine caruncle cell line (67) and that it can only be detected in the caruncle of one animal after 20 dpi (20). These findings support the idea that the low-virulence isolate has a lower replication rate that prevents the development of an exacerbated proinflammatory environment in the placenta, thus preventing structural and functional alterations and consequently abortion. We also corroborated the influence of the immune response on foetal death occurrence through clustering analyses. These analyses demonstrated that the progression of the infection until foetal death in caruncles resulted in an overexpression of genes enriched in biological processes related to the proinflammatory immune response. This corroborates the previously established hypothesis that propose that abortion is the consequence of an immune-mediated mechanism (3, 15–17, 21, 68). The overexpression of genes related to proinflammatory responses in heifers infected by the high-virulence isolate could be triggered by the expression of TFs, such as STAT1, STAT4, and NFKB1, which are necessary to mount an immune response against pathogens. In fact, the subversion exerted by *T. gondii* effectors on pathways governed by these TFs has been largely defined (69). In view of our results, it would be interesting to determine which *N. caninum* factors are involved in this type of host modulation and how the virulence of the parasite isolate influences this factor.

In the foetal part of the placenta, the results were markedly different from those observed in the caruncles. Both isolates induced a similar inflammatory response, being earlier and stronger in the animals infected with the high-virulence isolate. As commented previously for caruncles samples, these results contrast with the proposal of a higher immune stimulation by the Nc-Spain1H isolate (5), these discrepancies will be further investigated. Overall, we found a positive correlation between the RNA-seq data presented here and our previous qPCR results, except for the Nc-Spain1H-infected animals compared to the noninfected animals at 10 dpi on cotyledons, in which nonsignificant results were found, and this contrast deserves special attention. Although we found a high number of DEGs (2962) between the Nc-Spain1H-infected and noninfected animals in cotyledons, this result was not associated with an enrichment of any specific biological functions. However, this finding might reflect an early interaction of Nc-Spain1H with the host that subsequently enables local control against parasite replication. Nevertheless, despite the differences observed in the differential expression analyses, we found a high correlation between the transcriptome of animals infected with both isolates in cotyledons, which translates into a similar interaction of the Nc-Spain7 and Nc-Spain1H isolates with the foetal part of the placenta.

Deconvolution analyses from expressed genes demonstrated that caruncles infected with the high-virulence isolate (VF and NVF) presented a higher proportion of different immune populations, including monocytes, M1 macrophages, activated NK cells, CD8+ T cells, T follicular helper cells and activated mastocytes. Previous studies have shown the presence of an inflammatory infiltrate based on cells as CD3+, CD4+, CD8+, and γδTCR+, and NKp46+ in caruncles from animals infected at different stages of infection (15, 21, 68, 70). In addition, some of these cells have been associated to foetal death at early gestation (15, 21, 68). These works have also showed marked differences in the infiltration of immune cell populations depending on the stage of gestation at which infection occurs. Therefore, focusing on mid-gestation, in agreement with previous works, CD4 T cells would not to be responsible for the transcriptomic profiles described here (15). However, and in contrast to our results Cantón et al. (2014) (15) did not observe differences in the CD8+ or macrophages scores between challenged dams and control animals at mid-gestation. However, we first showed by immunohistochemistry that the animals infected with the Nc-Spain7 isolate displayed a higher proportion of T lymphocytes and phagocytic cells in placental tissues, which was more accentuated at 20 dpi (5). Subsequently, using flow cytometry, we detected an increase in CD8+ T cells in peripheral blood mononuclear cells from the heifers infected with the Nc-Spain7 isolate at 9, 13 and 20 dpi (71). CD8+ T cells are involved in the recognition and killing of cells infected with intracellular pathogens. Thus, the CD8+ increase would be an attempt to reduce parasite multiplication as a consequence of the higher invasion and replication of the Nc-Spain7 isolate. However, CD8+ increases could enhance tissue damage *in vivo* due to cytotoxic effects (72). In addition, as in this study, an *in vitro* study with bovine monocyte-derived macrophages showed that *N. caninum* infection induces macrophage polarization towards the M1 phenotype (6). Thus, we hypothesized that these cell populations could be responsible for the exacerbated immune response and tissue damage that occurs in the placenta during infection with the high-virulence isolate as has also been suggested for infections at early gestation (15, 21, 68). On the other hand, it is known that mediators from mast cells were also increased in the Nc-Spain7-infected heifers, playing a key role in inflammation and in the pathogenesis of other protozoan parasitic disease (73, 74), but to our knowledge, there are no studies about its importance in bovine neosporosis, and this issue should be investigated since there are some studies in humans that relate these cells to pregnancy disorders (56, 75). In addition, as described for *T. gondii* and *N. caninum*, some of these immune populations can be hijacked by the parasite, which can use them as a Trojan horse to spread systemically, cross biological barriers and reach immune privileged organs (53, 76). In fact, previous experiments performed with human dendritic cells infected with different *N. caninum* isolates have shown significant variations in their capacity to induce migration across membranes (77). In this sense, our previous work showed that the high-virulence isolate reaches the cotyledons earlier (at 10 dpi) than the low-virulence isolate, indicating that Nc-Spain7 has the ability to spread more efficiently to the foetal part (5, 20). In relation to this, we should note the strong transcriptomic induction of genes related to the epithelial mesenchymal transition in caruncles from the heifers infected with the high-virulence isolate at 10 dpi (**Figure 3A**). This transition may be advantageous for the Nc-Spain7 isolate, enhancing its invasive cellular capabilities and favoring transmission access to the foetal part of the placenta, as activation of epithelial mesenchymal transition causes defects in epithelial cells, loss their apical-basal polarity and cell−cell junctions and increased cell motility and cell invasion (78). Remarkably, M2 macrophages had a higher representation in caruncles from the animals infected with the low-virulence isolate than those from the high-virulence isolate-infected animals. In cattle, under physiological conditions, macrophages undergo M2 differentiation over the gestation period (79). This phenotype of macrophages is responsible for the normal microenvironment at the maternal–foetal interface (80) and is involved in anti-inflammatory responses (81). In fact, any bias in the M1/M2 macrophage balance may lead to pregnancy disorders (82, 83). This increase in M2 macrophages in the heifers infected with the Nc-Spain1H isolate could be key to preventing abortion, and given their role in anti-inflammatory responses, their presence could explain the lack of immune stimulation that we observed in the caruncles infected with the low-virulence isolate. In a context with a prevailing presence of M2 macrophages in the placenta, an anti-inflammatory environment would be promoted, thus counteracting the proinflammatory phenotype that a parasitic infection would trigger. This phenomenon would maintain the delicate immune balance in the placenta that ensures the maintenance of pregnancy (48). In line with this finding, previous studies carried out with *T. gondii* have shown that strain virulence determines the polarization of macrophages (84), and considering our findings, it would be reasonable to extrapolate this situation to *N. caninum*. In fact, the discovery of parasite factors that govern such macrophage polarization in different isolates could lay the foundation for the development of effective measures to prevent *N. caninum*-induced abortion. This polarization was not observed in the Nc-Spain1H-infected cotyledons, although studies focused on foetal macrophages are lacking. However, according to a recent study using term placentas, foetal and maternal macrophages are different and display different phenotypes (85). This study raises interesting questions about the phenotype displayed by foetal macrophages during gestation.

## 5 Conclusions

This study shows that isolates of different virulence differ in their interaction with the host placenta at early stages of infection, which could mediate the outcome of the infection. The high-virulence isolate triggers a proinflammatory immune response as early as 10 dpi that increases later in the infection. However, the low-virulence isolate does not activate such an inflammatory response, and there were even indications of inhibition, which could facilitate its transmission without causing intense damage to the caruncle. The exacerbated immune response triggered by the high-virulence isolate could be a key contributor to foetal death as previously suggested. The transcriptional changes induced by both isolates at the foetal part were more similar, and both triggered a proinflammatory response, although it was stronger and earlier in heifers infected with the high-virulence isolate. These results indicate that the tight modulation of the host response on the maternal side, the caruncle, may be decisive in determining the outcome of the infection; however, once the parasite reaches the fetus, this regulation may not be as important.

## Data availability statement

The datasets presented in this study can be found in online repositories. The names of the repository/repositories and accession number(s) can be found below: PRJEB58430 (European Nucleotide Archive-ENA).

## Ethics statement

The animal study was reviewed and approved by Animal Research Ethics Committee of the Principado de Asturias, Spain (reference number PROAE 25/2016).

## Author contributions

AR-P, LO-M, MC and PH conceived the study participated in its experimental design. IP-F, LR-S and PH prepared all samples for transcriptome sequencing. AR-P and MC develop all bioinformatics and statistical analyses. PH and MC wrote the manuscript with interpretation of results and discussion inputs from AR-P, EC-F, IP-F and LO-M. All authors contributed to the article and approved the submitted version.
