# Supplementary material for: Whole-transcriptome analysis reveals virulence-specific pathogen−host interactions at the placenta in bovine neosporosis
Source: Front Immunol. 2023 Jul 14;14:1198609. doi: 10.3389/fimmu.2023.1198609 (PMC10380943; doi:10.3389/fimmu.2023.1198609)
Supplement: Supplementary file 1 [file DataSheet_1.pdf]

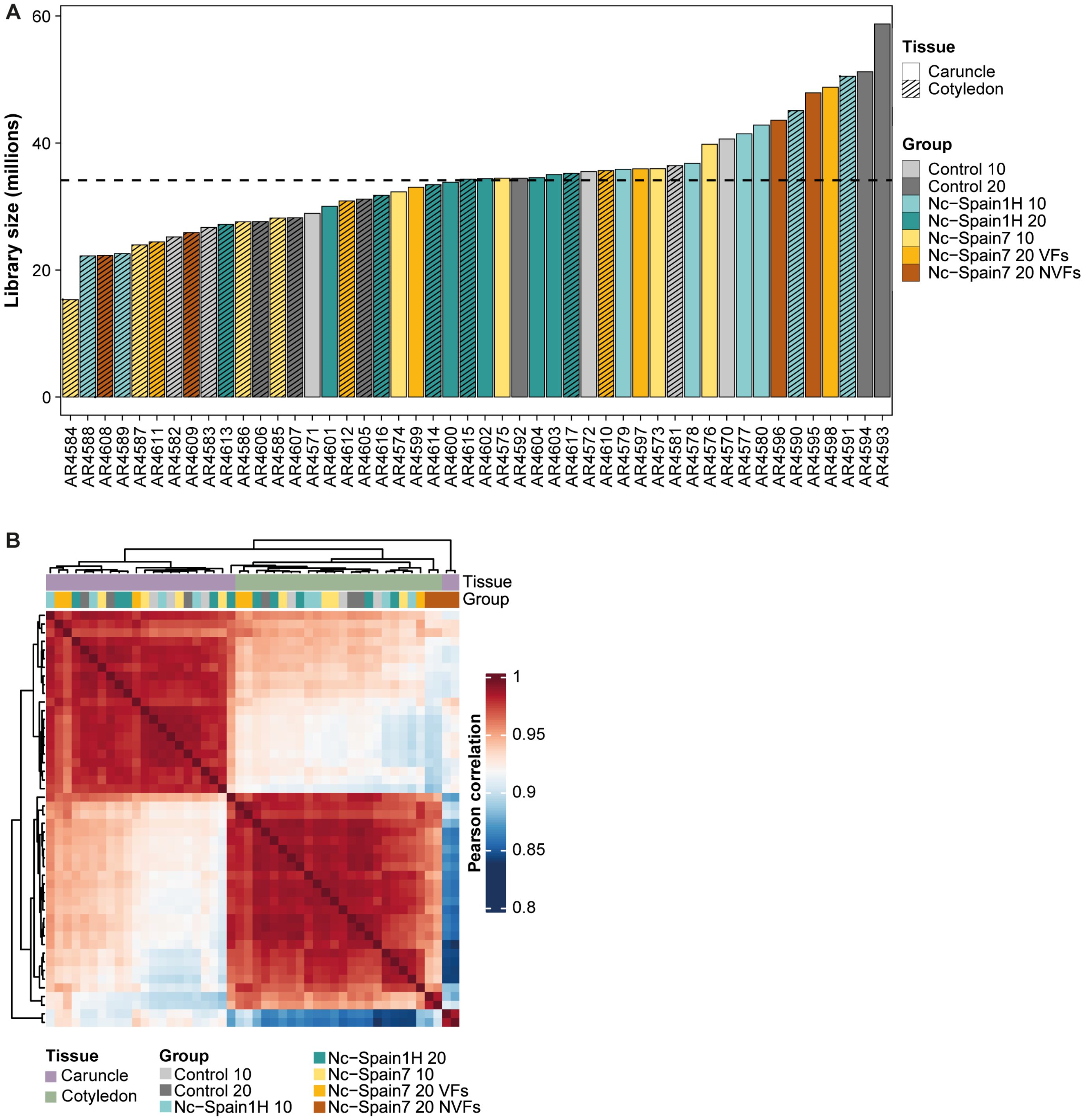

Supplementary Figure 1. Quality control of RNA-Seq data. (A) Bar plot represents the library size of each RNA-Seq sample. Heifer groups are represented by colours, and tissues are represented by patterns as indicated in the figure legend. The dashed line represents the mean library size. (B) Hierarchical clustering heatmap of Pearson correlation coefficients between caruncle (purple) and cotyledon (green) samples infected by high- and low-virulence *N. caninum* isolates.

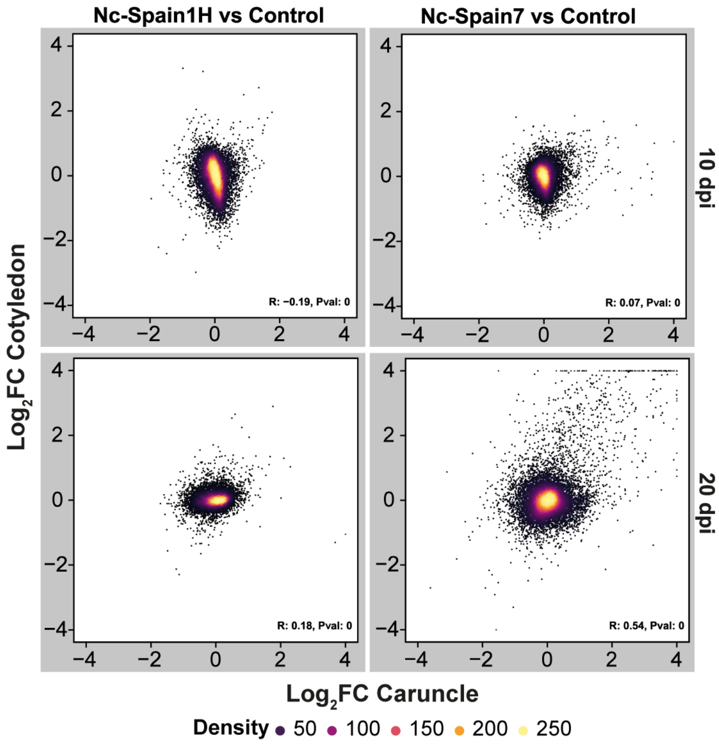

Supplementary Figure 2. Correlations of log 2-fold change values between *N. caninum* isolate-infected and noninfected animals. Dot plots represent the correlation between log2FC in caruncle and cotyledon of the Nc-Spain1H (left)- and Nc-Spain7 (right)-infected samples compared to the control samples at 10 (upper) and 20 dpi (lower). Each dot represents the log2FC in each gene. The colour gradient represents the density of genes from purple (low density) to yellow (high density).

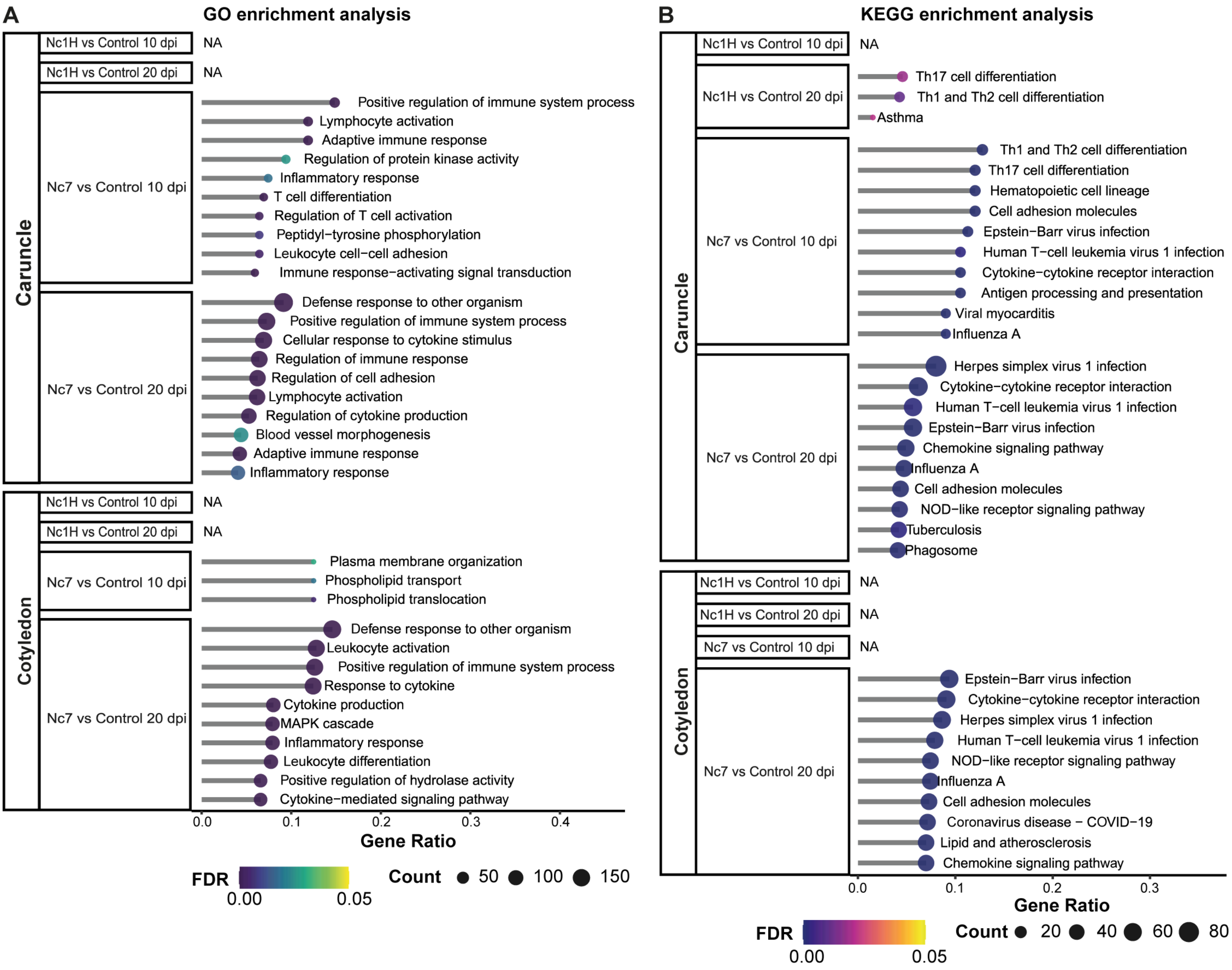

Supplementary Figure 3. Functional analysis across *N. caninum* isolate-infected and noninfected animals in placentomes. (A). Dot plot of GO enrichment analysis using DEGs between the *N. caninum* isolate-infected samples and the control samples in the caruncle and cotyledon. The top 10 terms with higher gene ratios are represented. Adjusted P values are represented by a colour gradient from blue (more significant) to yellow (low significant). (B). Dot plot of KEGG enrichment analysis using DEGs between the *N. caninum* isolate-infected samples and the control samples in the caruncle and cotyledon. The top 10 terms with higher gene ratios are represented. Adjusted P values are represented by a colour gradient from purple (more significant) to yellow (low significant). Enriched terms are ordered by gene ratio. Dot size represents the number of DEGs in each enriched term. Nc1H: Nc-Spain1H-infected animal. Nc7: Nc-Spain7-infected animals.

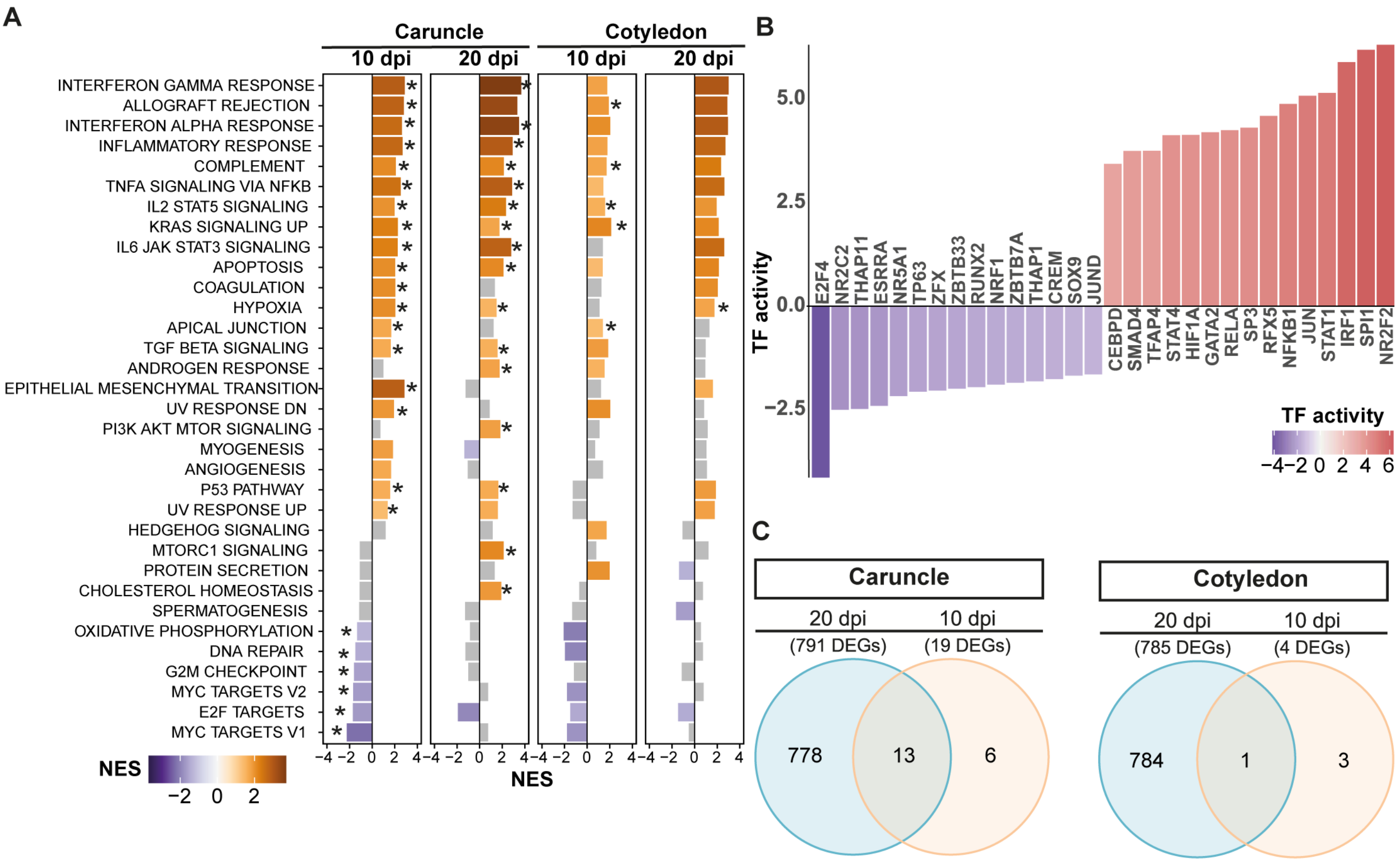

Supplementary Figure 4. Transcriptomic differences between high- and low-virulence isolate-infected placental samples. (A). Bar plots show the normalized enrichment scores (NESs) of GSEA for hallmark gene sets using the stat value from differential expression analysis. NES are represented by a colour gradient from purple (upregulated in the Nc-Spain1H-infected samples) to orange (upregulated in the Nc-Spain7-infected samples). Grey bars represent nonsignificant hallmark gene sets (adjusted p value > 0.1) * indicate isolate-specific pathways. (B). Bar plot represents the top 15 TF activities between the Nc-Spain1H- and Nc-Spain7-infected samples. The colour gradient represents the TF activity value from blue (more activity in the Nc-Spain1H-infected samples) to red (more activity in the Nc-Spain7-infected samples). (C). Venn diagrams show the overlap of DEGs between the Nc-Spain7- and Nc-Spain1H-infected samples at 10 and 20 dpi in caruncle (left) and cotyledon (right) tissues.

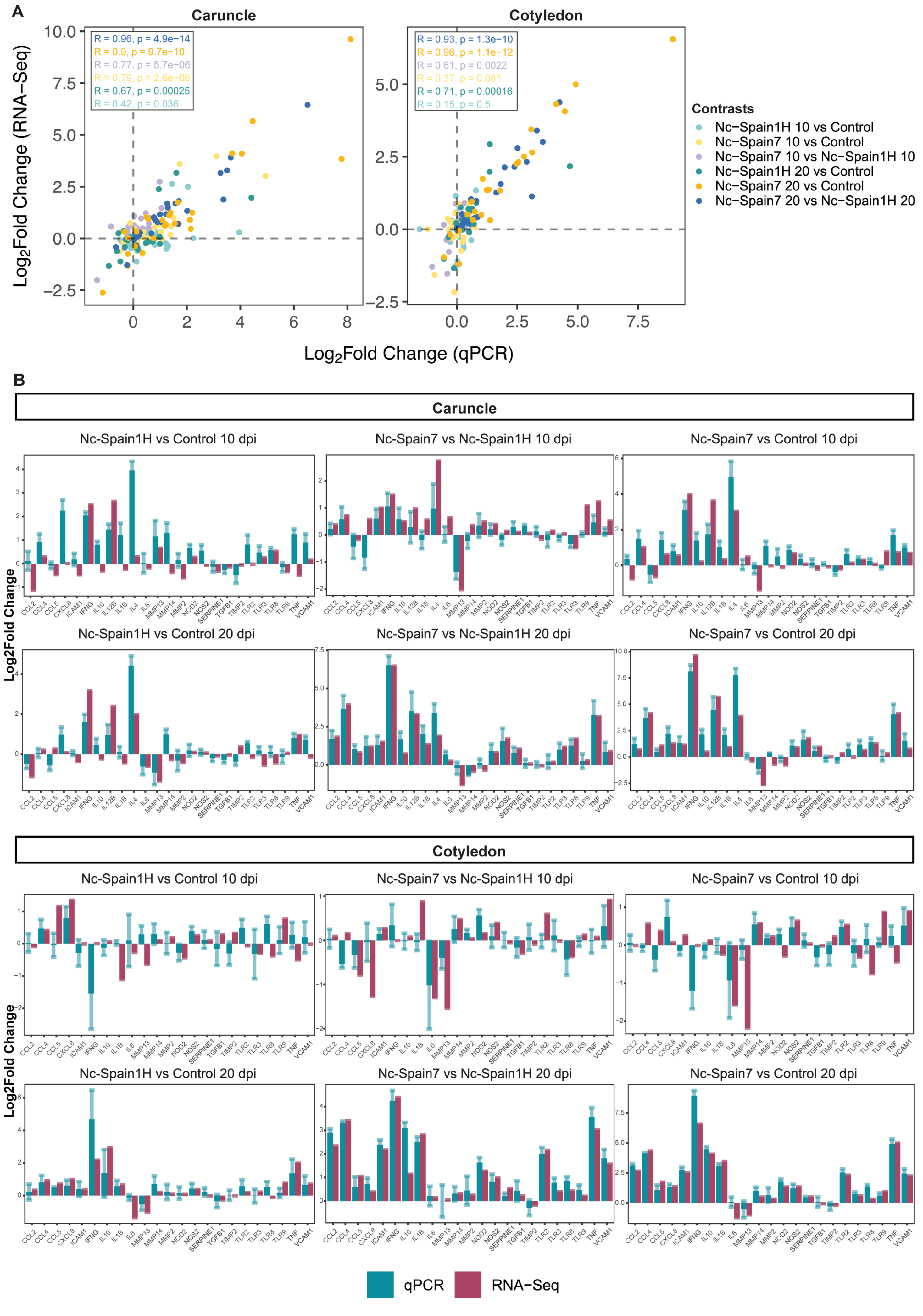
A
Caruncle
Cotyledon
R = 0.96, p = 4.9e−14
R = 0.9, p = 9.7e−10
R = 0.77, p = 5.7e−06
R = 0.79, p = 2.6e−06
R = 0.67, p = 0.00025
R = 0.42, p = 0.036
R = 0.93, p = 1.3e−10
R = 0.96, p = 1.1e−12
R = 0.61, p = 0.0022
R = 0.37, p = 0.081
R = 0.71, p = 0.00016
R = 0.15, p = 0.5
Log2Fold Change (RNA−Seq)
Log2Fold Change (qPCR)
Contrasts
Nc−Spain1H 10 vs Control
Nc−Spain7 10 vs Control
Nc−Spain7 10 vs Nc−Spain1H 10
Nc−Spain1H 20 vs Control
Nc−Spain7 20 vs Control
Nc−Spain7 20 vs Nc−Spain1H 20
B
Caruncle
Nc-Spain1H vs Control 10 dpi
Nc-Spain7 vs Nc-Spain1H 10 dpi
Nc-Spain7 vs Control 10 dpi
Nc-Spain1H vs Control 20 dpi
Nc-Spain7 vs Nc-Spain1H 20 dpi
Nc-Spain7 vs Control 20 dpi
Cotyledon
Nc-Spain1H vs Control 10 dpi
Nc-Spain7 vs Nc-Spain1H 10 dpi
Nc-Spain7 vs Control 10 dpi
Nc-Spain1H vs Control 20 dpi
Nc-Spain7 vs Nc-Spain1H 20 dpi
Nc-Spain7 vs Control 20 dpi
Log2Fold Change
CCL2 CCL4 CCL5 CXCL8 ICAM1 IFNG IL10 IL12B IL1B IL4 IL6 MMP13 MMP14 MMP2 NOD2 NOS2 SERPINE1 TGFB1 TIMP2 TLR2 TLR3 TLR8 TLR9 TNF VCAM1
qPCR
RNA−Seq

Supplementary Figure 5. qPCR validation of RNA-Seq data. (A). Correlation between log2 fold change of RNA-Seq (y-axis) and qPCR (x-axis) on selected genes related to immunity between the *N. caninum* isolate-infected samples compared to the control samples and between high- and low-virulence isolate-infected samples at 10 and 20 dpi in caruncle (left) and cotyledon (right). Each point represents the value of log2-fold change from RNA-Seq and qPCR in a specific gene. Colours are specific from the contrast. (B). Bar plots represent the log2-fold fold change value from both techniques, red bars for RNA-Seq and blue bars for qPCR separately for each contrast in caruncle (upper) and cotyledon (lower).
